# Assessing the feasibility of confocal laser endomicroscopy in solitary pulmonary nodules for different part of the lungs, using either 0.6 or 1.4 mm probes

**DOI:** 10.1371/journal.pone.0189846

**Published:** 2017-12-21

**Authors:** Tidi Hassan, Luc Thiberville, Christophe Hermant, Samy Lachkar, Nicolas Piton, Florian Guisier, Mathieu Salaun

**Affiliations:** 1 Department of Respiratory Care, Thoracic Oncology, and Respiratory Intensive Care & CIC-CRB U1404, Rouen University Hospital, Rouen, France; 2 Department of Medicine, Faculty of Medicine, Universiti Kebangsaan Malaysia, Cheras, Malaysia; 3 Normandie Univ, UNIROUEN, LITIS, Quant.I.F, Rouen, France; 4 Service de Pneumologie-Allergologie, Hôpital Larrey, CHU de Toulouse, Université de Toulouse III (Paul Sabatier), Toulouse, France; 5 Department of Pathology & Cytology, Rouen University Hospital, Rouen, France; European Organisation for Research and Treatment of Cancer, BELGIUM

## Abstract

**Background:**

Malignant solitary pulmonary nodules (SPN) have become more prevalent, with upper lobes predilection. Probe-based confocal laser endomicroscopy (pCLE) provides *in-vivo* imaging of SPN. However, the stiffness of the 1mm confocal probe (AlveoFlex) causes difficult accessibility to the upper lobes. A thinner 600μm probe designed for bile duct exploration (CholangioFlex) has the potential to reach the upper lobes.

**Objectives:**

To examine the accessibility of malignant SPNs in all segments of the lungs using either the 0.6mm or 1.4 mm probe and to assess the quality and inter observer interpretation of SPN confocal imaging obtained from either miniprobes.

**Methods:**

Radial(r)-EBUS was used to locate and sample the SPN. *In-vivo* pCLE analysis of the SPN was performed using either CholangioFlex (apical and posterior segments of the upper lobes) or AlveoFlex (other segments) introduced into the guide sheath before sampling. pCLE features were compared between the two probes.

**Results:**

Fourty-eight patients with malignant SPN were included (NCT01931579). The diagnostic accuracy for lung cancer using r-EBUS coupled with pCLE imaging was 79.2%. All the SPNs were successfully explored with either one of the probes (19 and 29 subjects for CholangioFlex and AlveoFlex, respectively). A specific solid pattern in the SPN was found in 30 pCLE explorations. Comparison between the two probes found no differences in the axial fibers thickness, cell size and specific solid pattern in the nodules. Extra-alveolar microvessel size appeared larger using CholangioFlex suggesting less compression effect. The kappa test for interobserver agreement for the identification of solid pattern was 0.74 (*p* = 0.001).

**Conclusion:**

This study demonstrates that pCLE imaging of SPNs is achievable in all segments of both lungs using either the 0.6mm or 1.4mm miniprobe.

## Introduction

Probe-based confocal laser endomicroscopy (pCLE) is an emerging technology that complements standard white-light bronchoscopy to provide *in-vivo* and real-time imaging of the lungs [1–3]. Using the well-established principles of confocal microscopy and fibreoptics, pCLE using the Cellvizio® device (Mauna Kea Technologies, Paris, France) enables ‘optical biopsy’ of the distal lung by introducing a confocal miniprobe (AlveoFlex®) with an outer diameter of 1.4 mm that can be introduced into the 2mm working channel of a flexible bronchoscopy (Alveoscopy) [2].

Previously, in-vivo confocal microscopic imaging of the distal lung fluorescent structures in response to 488nm blue light excitation resulted in the description of pCLE imaging of the normal alveolar ducts, extra-alveolar microvessels and alveolar cells in both non-smoking and actively smoking subjects [4]. There are growing literature related to the application of pCLE in pulmonary diseases including pulmonary alveolar proteinosis [5], diffuse emphysema [6], amiodarone-induced pneumonitis [7] and acute lung allograft rejection [8].

Solitary pulmonary nodules (SPN) have become more prevalent and have resulted in diagnostic challenges in clinical practice, especially due to the increasing reports of peripheral lung adenocarcinoma [9, 10]. With the help of endoscopic techniques such as navigation bronchoscopy, exploration of SPNs with pCLE may aid diagnostic work-up. Our initial observation has demonstrated that SPNs explored with pCLE revealed solid patterns in which the normal alveolar network is not recognizable and associated with area of increased density [11].

However, most primary malignant SPNs are located in the upper lobes, especially on the right lung with sixty percent located in the periphery of the lungs [12, 13]. In a previous study, we have shown that the upper and posterior segments of both lungs appear difficult to reach using the AlveoFlex® miniprobe because of the stiffness of the tip [4]. Therefore, thinner and more flexible miniprobes are necessary to reach difficult-to-access SPNs, especially in the upper lobes.

The CholangioFlex® miniprobe, which has been designed for bile duct explorations is one of the smallest pCLE miniprobe. With an outer diameter of 0.6 μm, the CholangioFlex® miniprobe could be potentially applied to image the distal lung. In this study, we hypothesised that pCLE is accessible for all malignant SPNs located in different part of the lungs and the same information could be obtained from images recorded using either the AlveoFlex® and CholangioFlex® miniprobes. The subjects recruited for this study were part of the prospective controlled clinical trial (NODIVEM:NCT01931579) that assessed pCLE in adjunction to navigational bronchoscopy in peripheral lung nodules. The authors confirm that all ongoing and related trials for this intervention are registered. The objectives of this study were to examine the accessibility of malignant SPNs in all segments of the lungs using either probes and to compare the performances of the probes in regards to pCLE imaging.

## Material and methods

### Subjects

**Fig 1:**
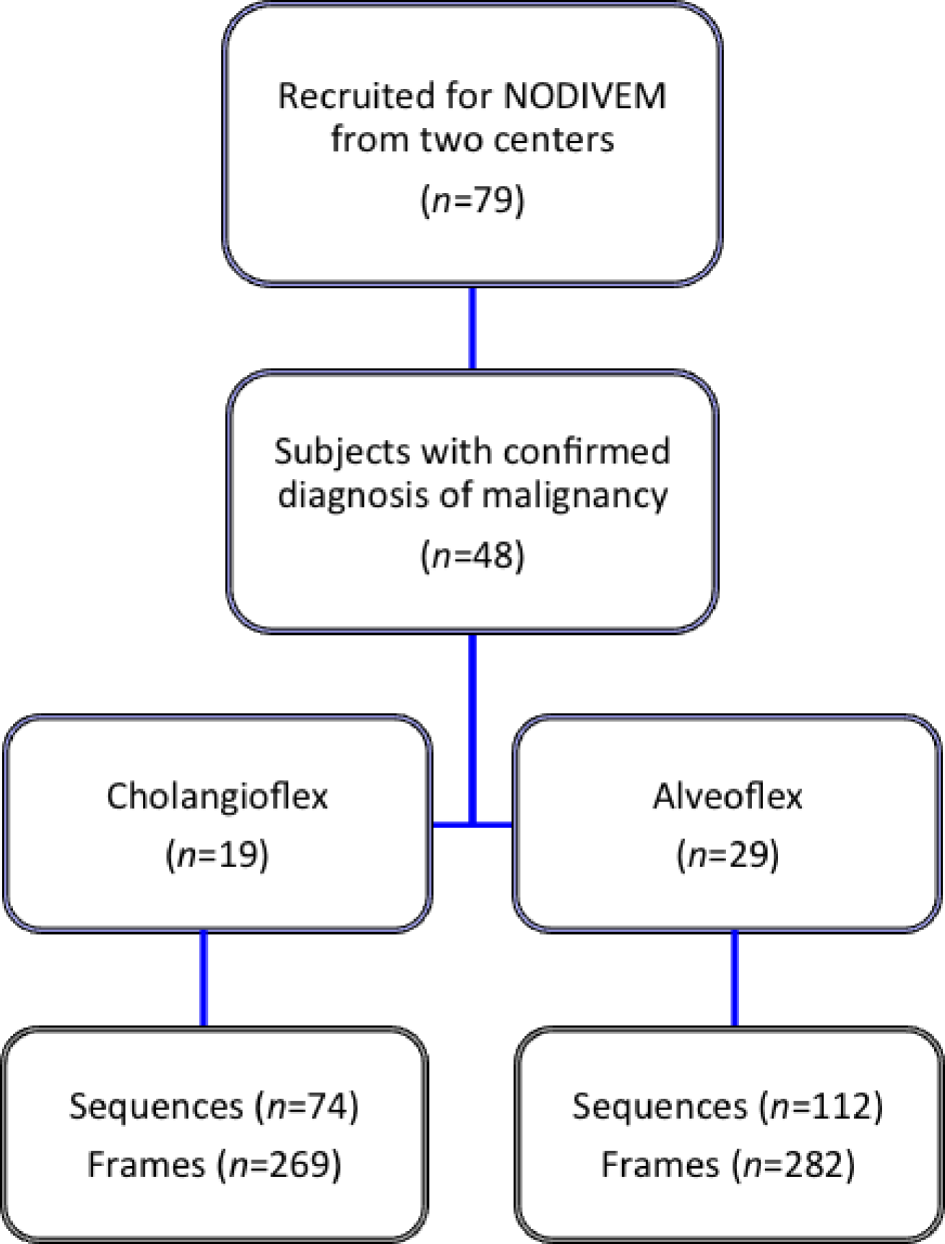
Flow chart of the study. Subjects were recruited from either one of the two centers of the NODIVEM study. Inclusion criteria in the NODIVEM study included a final diagnosis of malignant SPN with histopathology confirmation and successful location of the SPN with r-EBUS. All of the patients signed a written informed consent before the pCLE procedure, which was performed with topical lidocaine but without conscious sedation.

This is a pilot, exploratory study to conduct a comparative analysis of pCLE with the AlveoFlex® and CholangioFlex® minprobes, in which subjects were selected from the cohort prospectively enrolled in the NODIVEM (Assessment of Probe Based Confocal Laser Endo-microscopy for *In-vivo* Diagnosis of Peripheral Lung Nodules and Masses) trial (S1 and S2 Files). This trial involved three centres (Rouen University Hospital (LT), Toulouse University Hospital (CH), and St. Etienne University Hospital, France). pCLE using the CholangioFlex® miniprobe was not performed in St. Etienne University Hospital and subjects recruited into the NODIVEM trial from this centre were excluded in this study. For this ancillary study, inclusions started on November 8^th^, 2012, after the authorization to use the CholangioFlex® probe (CPP Nord-Ouest I, number *CPP 2011/030 –Amdt n°1*), and ended on October 9^th^, 2014. Patients were followed up during 6 months (Fig 1). The NODIVEM study was registered in ClinicalTrials database (ClinicalTrials.gov identifier: NCT01931579) on July 29^th^, 2013, nine months after the inclusion of the first patient, due to administrative processing delay. The study was approved by the ethical committee CPP Nord-Ouest I, number *CPP 2011/030*, on November 14^th^, 2011, with amendment for the use of CholangioFlex® probe on July 19th, 2012.

### *In-vivo* real time alveolar microscopic imaging

The confocal miniprobes used for this study included standard, commercially available AlveoFlex® miniprobe, and modified CholangioFlex® miniprobe with a depth of focus of 0–50 μm which is identical to those of the AlveoFlex® miniprobe (Mauna Kea Technologies, Paris, France).

In order to evaluate SPNs with pCLE, we developed a technique based on pCLE of the distal lung (Alveoscopy) [4]. This method uses peripheral r-EBUS coupled with a 1.95mm external sheath and virtual navigation to locate the SPN. Briefly, the navigational software (superDimension^TM^) was used to determine the endobronchial path in which the bronchus of interest is aligned in three dimensions towards the SPN. The distance between the closest subsegmental bronchi visualized and the target was measured. We then introduced the 20 MHz r-EBUS probe and 1.4 mm external sheath into the working channel of a 4mm flexible bronchoscope (MP60 model; Olympus, Tokyo, Japan) to reach the SPN [14].

**Fig 2:**
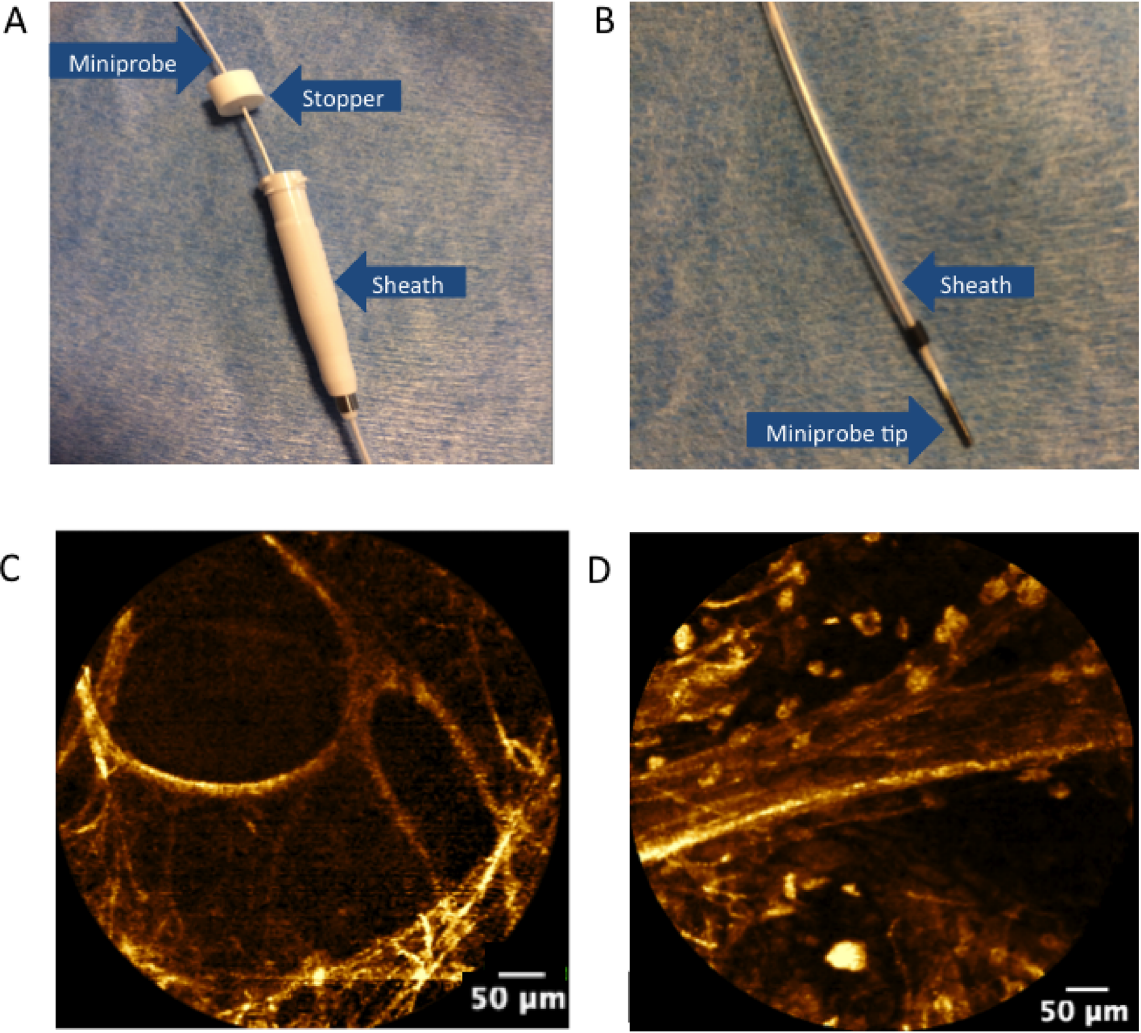
probe-based confocal endomicroscopy probe in the r-EBUS guide sheath, and examples of alveolar pCLE imaging. *A*, A stopper is first used to determine that the tip of the confocal miniprobe. *B*, coincides with the distal tip of the external guide sheath. C&D. Endomicroscopic images of the distal lung (autofluorescence, 488 nm excitation, Cell-vizio Lung-488) showing: *C*, normal axial elastic backbone of an alveolar duct in a non-smoking subject; and *D*, a blood vessel with cellular infiltration by fluorescent cells, presumably alveolar macrophages.

Using the Cellvizio®-Lung device with 488 nm excitation (Mauna Kea Technologies, Paris, France), either the AlveoFlex® or CholangioFlex® miniprobes was then introduced into the sheath in order to obtain *in-vivo* pCLE images of the SPN. The AlveoFlex® miniprobe was used to evaluate SPNs in the lingular, middle and lower lobes in addition to the anterior lobes of the upper lobes. The CholangioFlex® miniprobe was used to image the apical and posterior segments of the upper lobes or other subsegments that are not reachable with the AlveoFlex®. A stopper was positioned on the confocal miniprobe to ascertain that the tip of the confocal miniprobe coincides with the tip of the external guide that guides the r-EBUS (Fig 2A and 2B).

Imaged areas were subsequently sampled with both cytology brushings and biopsy forceps. The procedures were performed by two endoscopists in the two centers under local anesthesia.

### Imaging data analysis

**Fig 3:**
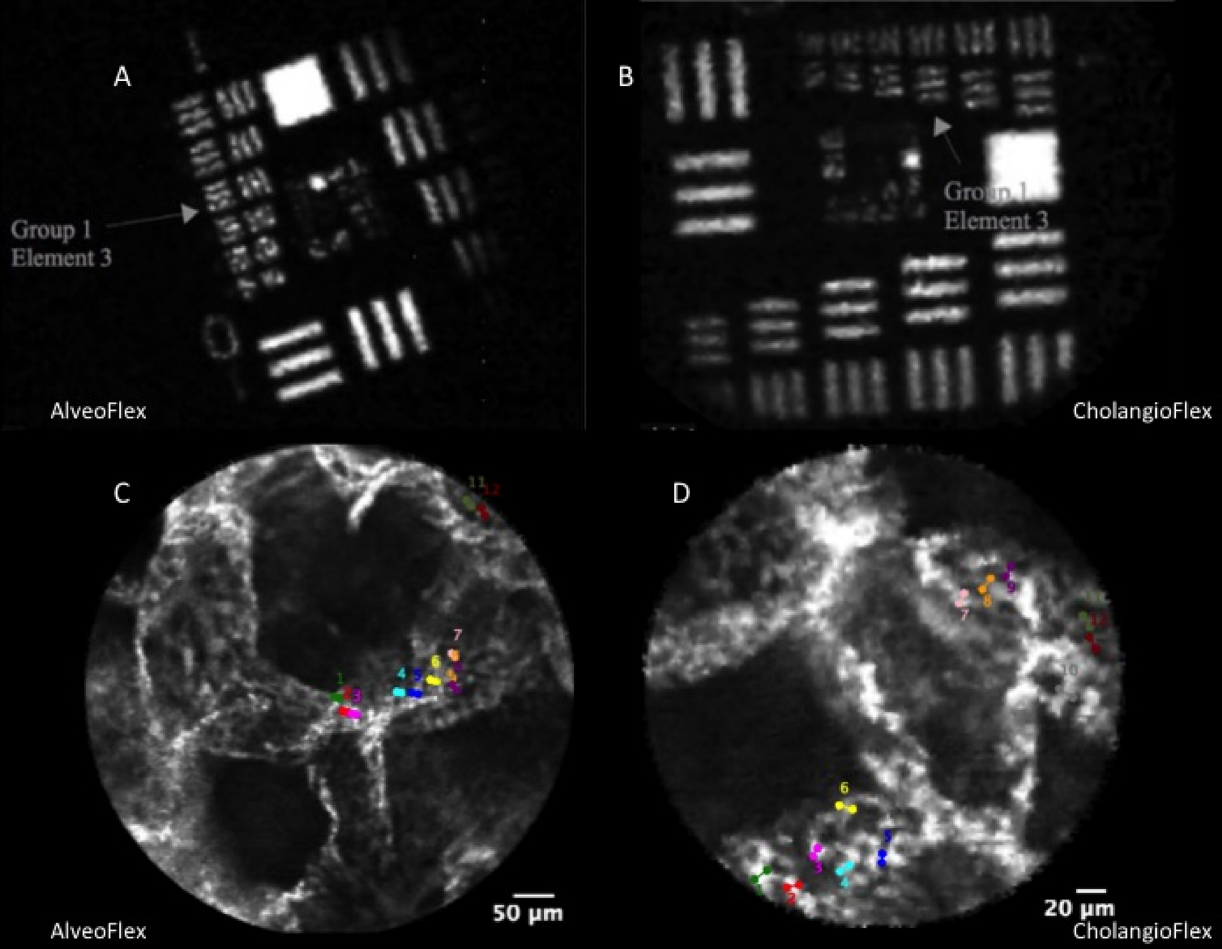
A&B: Image of the USAF 1951 pattern obtained under 488 nm excitation with AlveoFlex (A) and CholangioFlex (B) confocal miniprobes. Element 3 group 1 with frequency 151 lp/mm can be clearly identified, which corresponds to a bar width of 3.3 μm. These results are consistent with the design specification for resolution of CholangioFlex Miniprobes (3.5 μm). C&D: In order confirm reliability of measures with the two different probes, we imaged *ex-vivo* a well-preserved lung sample, fixed in glutaraldehyde at 20 cmH2O pressure, with the AlveoFlex (C) and CholangioFlex (D). On the images obtained with the two different probes, diameters of twelve pulmonary alveolar capillaries were measured, and the comparison showed no difference between the two probes.

The CholangioFlex® and Alveoflex® were used to image the same optical phantom in order to ensure the reliability of comparisons of measurements with the two probes (Fig 3). Furthermore, in the perspective of pulmonary imaging, the two probes were used to measure, *ex-vivo*, the pulmonary capillaries of a well-preserved lung sample, fixed in glutaraldehyde at 20 cmH2O pressure (Fig 3).

The median diameters of twelve capillaries, measured with CholangioFlex (median ± [interquartile range]: 8.7 ± [7.6–9.8] μm) and AlveoFlex (7.1 ± [6.4–8.9] μm), were not different (p = 0.16; Mann-Whitney test).

In order to analyse the pCLE images, sequences were examined frame by frame. Frames were selected based on

1. Recognition of identifiable alveolar network and its components including cells and blood vessels as previously described (Fig 2B and 2C) (3) and/or;
2. Recognition of a ‘solid pattern’, defined as areas in which normal alveolar network was not recognizable and associated with a dense appearance
3. Frames that appeared as pCLE imaging of the bronchi were excluded

These frames were saved as native (.mkt files) images in which the analysis was performed by two observers (LT and TH) using the dedicated Medviewer® 1.1 software (Mauna Kea Technologies, Paris, France). Measurements for long and short axis diameters of the alveoli, cell size and blood vessel size were assessed according to its greatest dimension. For axial fibre thickness, 3 to 5 fibres were measured from a representative image.

The images were categorized as having

1. A solid pattern with less than 50 or more than 50 percent of the field view
2. Distorted alveolar network in which the alveoli structures were intact but the size and shape were irregular
3. Destroyed alveolar network in which alveolar individual fibres were still present but the alveoli structures were not recognizable
4. Increased density due to increased number of alveolar fibres but not associated with a solid pattern
5. The presence of fluorescent cells
6. The presence of cell clustering
7. The presence of blood vessels

### Statistics

The descriptive analyses will be presented as mean +/- standard deviation (SD) or number (percentage). The frequencies of the pCLE features were compared between the pCLE sequences obtained from the CholangioFlex® or AlveoFlex® miniprobes using the Fisher’s exact test. For normally distributed variables, the unpaired 2-tailed *t*-test was used. A two-sided *p* value <0.05 was considered to be significant. Interobserver agreement was assessed using the kappa (***κ***) coefficient. Statistical analyses were performed using SPSS version 20 (Inc, Chicago, IL).

## Results

### Subjects’ characteristics

79 subjects under the NOVIDEM Trial (NCT01931579) prospectively underwent pCLE imaging from May 1 2012 until October 2014 in two centres (Rouen and Toulouse University Hospital). No mild or serious adverse event such as bleeding, post-procedure infection or pleural complication occurred during the pCLE procedures. 48 subjects with a final diagnosis of malignant SPN were included for data analysis (Fig 1). The mean ± SD age of the subjects was 63.3 ± 11.6.

The percentage of lung cancers diagnosed with histopathological evidence obtained from biopsies using r-EBUS coupled with pCLE imaging in the 48 subjects was 79.2%. Adenocarcinoma was the most common final diagnosis (N = 24), accounting half of the cases whilst squamous cell carcinoma was diagnosed in 13 subjects (27%). Other final diagnoses included 2 carcinoid tumours, 2 neuroendocrine tumours, an undifferentiated large cell carcinoma, a lymphoma and four metastatic nodules.

### Accessibility

**Fig 4:**
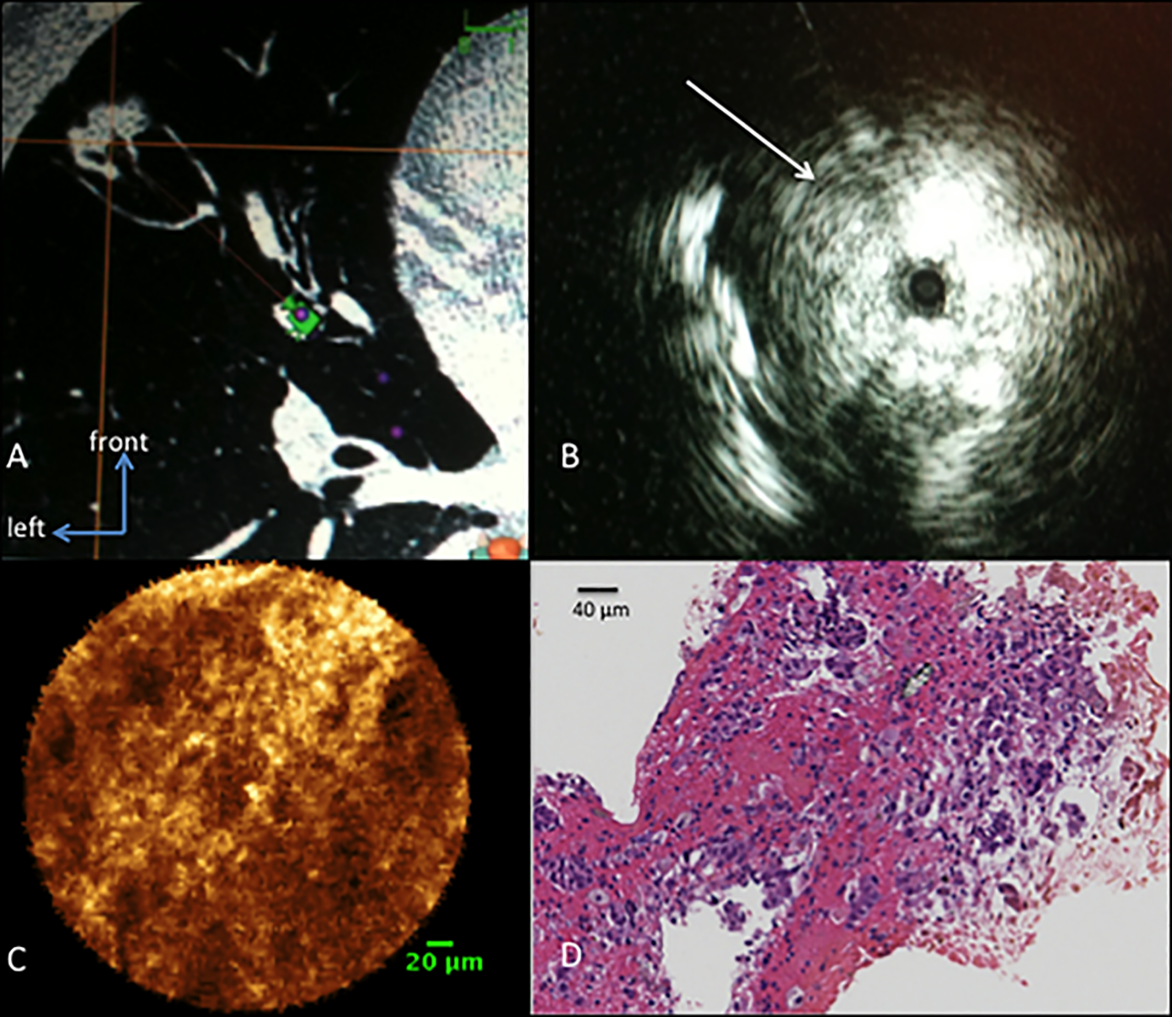
Example of one case with radiologic images of a solitary pulmonary nodule, a respectively r-EBUS image, the pCLE image and corresponding histology. A: 15 mm solitary pulmonary nodule of the lingula, located at 10 mm of the pleura. B: radial-EBUS signal in this nodule shows a tangential signal on the left part of the image (white arrow). C: pCLE image of this nodule shows a solid pattern on the whole field of view using the 0.6mm CholangioFlex® confocal miniprobe (scale bar: 20 μm). D: H&E staining of the biopsy performed during the procedure shows a pulmonary adenocarcinoma (Magnification x 40; scale bar: 40 μm).

The location of the SPNs is demonstrated in Table 1. Using r-EBUS, the mean size ± SD of the SPN was 21.2 ± 6.8 mm (range: 8–29). The SPNs were successfully explored with either one of the miniprobes once located with r-EBUS (Fig 4).

**Table 1:** Characteristics of SPNs imaged by AlveoFlex^®^ and CholangioFlex^®^ miniprobes.

Miniprobe	CholangioFlex^®^	AlveoFlex^®^	Total
Location of SPN			
*RUL (RB1*, *RB2)*	6	0	6
*RUL (RB3)*	0	2	2
*LUL (except for lingula)*	9	0	9
*RML*	1	9	10
*Lingula*	1	3	4
*RLL*	1	5	6
*LLL*	1	10	11

Abbreviations: SPN = solitary pulmonary nodule, RUL = right upper lobe, LUL = left upper lobe, RML = right middle lobe, RLL = right lower lobe, LLL = left lower lobe.

In one case involving the right middle lobe, the CholangioFlex® miniprobe was successfully utilised after we failed to reach the SPN using the AlveoFlex® miniprobe.

The mean ± SD pCLE imaging duration was 78 ± 53 (range: 10–226) seconds per patient with no significant difference between the two miniprobes (*p* = 0.149). 551 frames were selected from 186 sequences for analysis.

### Image analysis

**Fig 5:**
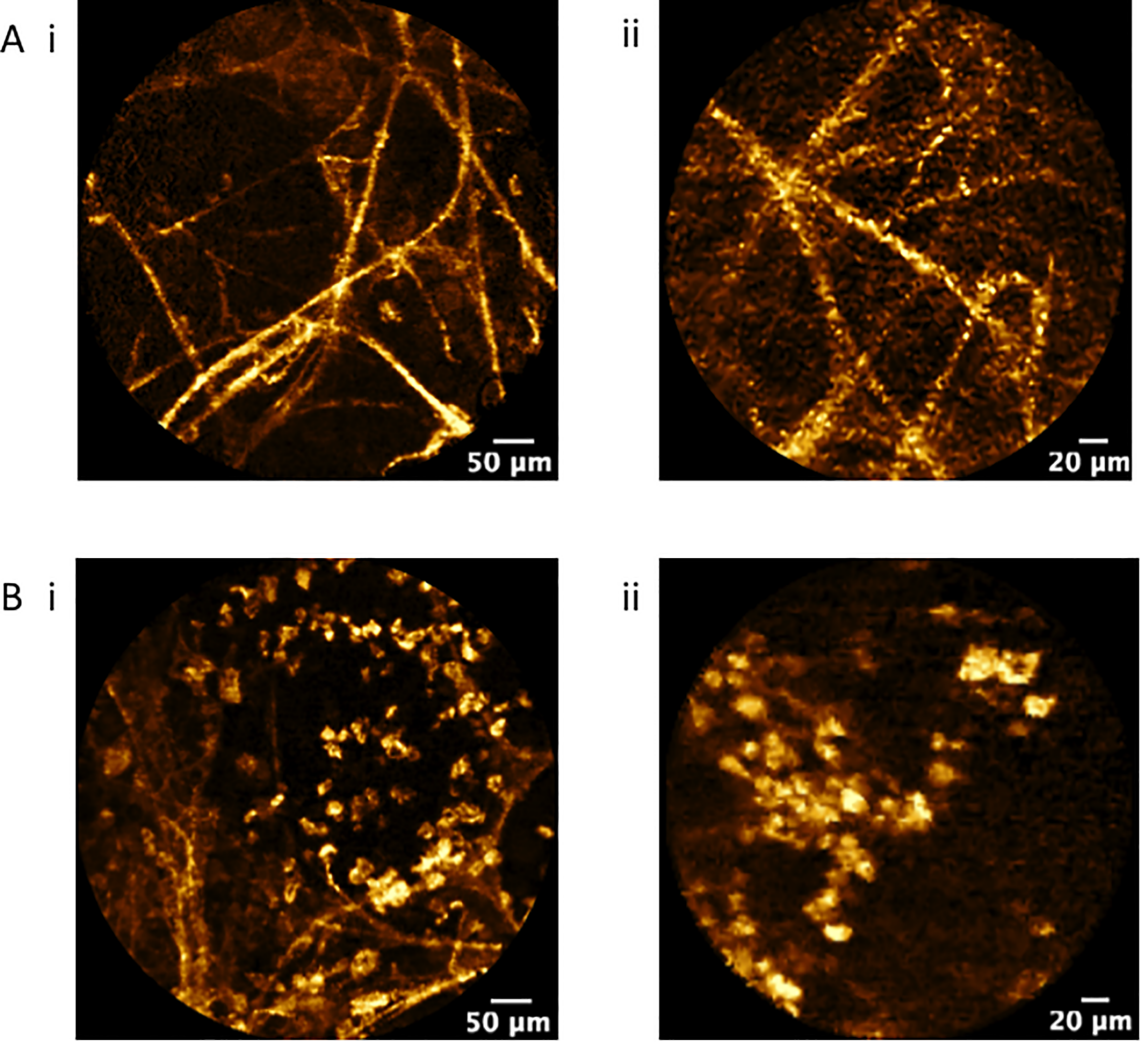
**Confocal fluorescence endomicroscopy of SPN (autofluorescence, 488 nm excitation, Cell-vizio Lung 488) demonstrating**: *A*, axial elastic backbone of an alveolar duct and *B*, cellular infiltration by fluorescent cells, presumably alveolar macrophages using (i) AlveoFlex® miniprobe and (ii) CholangioFlex® miniprobe.

The CholangioFlex® miniprobe revealed identifiable alveolar structure in 63%, compared to 69% with the AlveoFlex® miniprobe (*p* = 0.45). From these images, mean ± SD alveolar long-axis and short-axis diameter were 222.31 ± 50.89 μm vs 341.83 ± 86.92 μm and 155.54 ± 55.92 μm vs 208.94 ± 65.18 μm for the CholangioFlex® and AlveoFlex® miniprobes respectively. These differences were both statistically significant (*p* = 0.001 and p = 0.039). Axial fibre thickness had a mean ± SD of 18.57 ± 5.87 μm for the CholangioFlex® miniprobe and 14.6 ± 5.67 μm for the AlveoFlex® miniprobe (Fig 5A) (p = 0.058).

For cell size measurements, there was no difference between the two groups (mean ± SD of 22.30 ± 4.8 μm vs 26.1 ± 8.9 μm for the CholangioFlex® and AlveoFlex® miniprobes respectively (*p* = 0.45)) (Fig 5B). The frequency for the presence of cells was similar with no significant differences in both miniprobes (35% vs 27%) (*p* = 0.42). Cell clustering was also identified at similar frequency for both miniprobes (8% vs 7%)(*p* = 0.98).

Although there was no difference between the frequency of the presence of blood vessels, the difference between the vessel sizes measured was significant with a mean ± SD of 228.41 ± 152.27 μm and 82.96 ± 33.97 μm for the CholangioFlex® and AlveoFlex® miniprobes respectively (*p* = 0.014).

### *In-vivo* analysis of solid pattern

**Fig 6:**
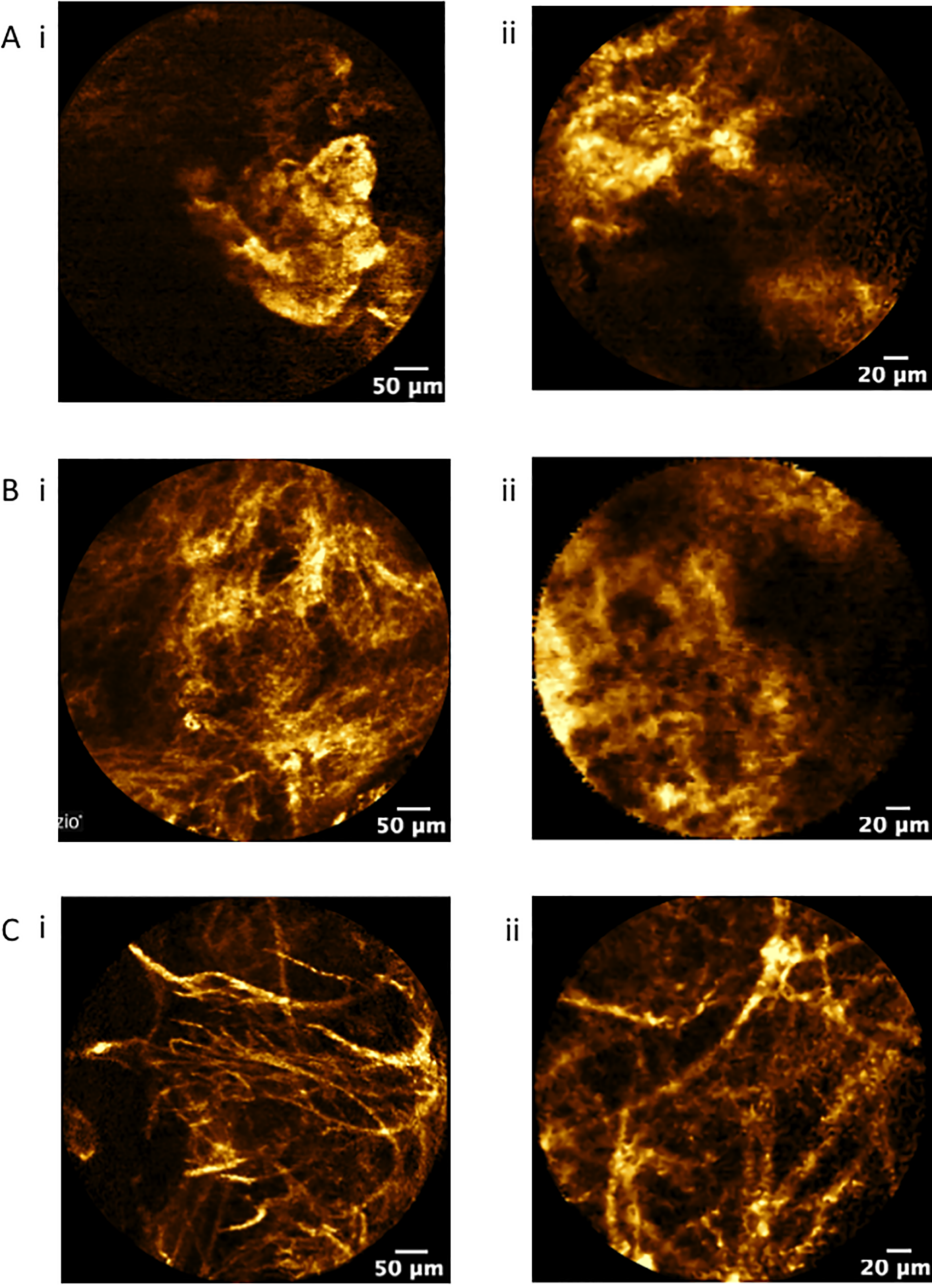
**Confocal fluorescence endomicroscopy of SPN (autofluorescence, 488 nm excitation, Cell-vizio Lung 488) demonstrating** *A*, solid pattern in less than 50% of the field view, and *B*, solid pattern in more than 50% of the field view and *C*, increased fibres in SPNs with confirmed adenocarcinoma using (i) AlveoFlex® miniprobe and (ii) CholangioFlex® miniprobe.

30 subjects had pCLE sequences that were identified to have solid pattern with 25 subjects graded as having a solid pattern with more than 50% of the total field view (Table 2) (Fig 6A and 6B). All of the images categorized as solid patterns were classified as either having distorted alveolar network (N = 28), destroyed alveolar network (N = 24), increased density (N = 24) and/or cellularity (N = 13). Similar rates were displayed for both the CholangioFlex® and AlveoFlex® miniprobes (Table 2). Fig 6C shows a representative of confocal images from two SPNs with destroyed alveolar network and increased fibres using either the AlveoFlex® or CholangioFlex® which were subsequently confirmed as adenocarcinomas.

**Table 2:** Characteristics of confocal images of SPNs by AlveoFlex and CholangioFlex miniprobes detected by at least one of the two observers.

	Total	CholangioFlex® (n, %)	AlveoFlex^®^ (n; %)	p-value
Solid pattern	30	13 (43)	17 (56)	0.55
No solid pattern	18	6 (33)	12 (67)	
*<50% of field view*	5	3 (60)	2 (40)	0.34
*>50% of field view*	25	12 (48)	13 (52)	
Alveolar distortion	28	13 (46)	15 (54)	0.37
No alveolar distortion	20	6 (30)	14 (70)	
Alveolar destruction	24	10 (42)	14 (58)	0.27
No alveolar destruction	24	9 (38)	15 (62)	
Increased density	24	8 (33)	16 (67)	0.56
No increased density	24	11 (46)	13 (54)	
Increased cellularity	13	6 (46)	7 (54)	0.74
No increased cellularity	35	13 (37)	22 (63)	

Fisher exact test

### Interobserver agreement

The κ coefficient for the descriptors is demonstrated in Table 3. The κ coefficient was very good for the identification of solid pattern in all subjects (0.74, *p* = 0.001), the CholangioFlex® (0.72, *p* = 0.03) and the AlveoFlex® (0.77, *p* = 0.03). The interobserver agreement observed for the presence of cells and blood vessels (κ coefficient>0.80) was almost perfect (0.99, *p* = 0.001). The identification of distorted versus destroyed alveoli was less robust (0.34 and 0.56, *p* = 0.17 and *p* = 0.001 respectively), while the agreement for either distorted or destroyed alveoli was good (0.82, p = 0.02).

**Table 3:** Interobserver agreement (*κ*).

Criteria	*κ* Total	95% CI	*κ* Cholangioflex	95% CI	*κ* Alveoflex	95% CI
Solid pattern	0.74	0.71–0.79	0.72	0.69–0.75	0.77	0.72–0.82
Distorted alveoli	0.34	0.23–0.41	0.36	0.26–0.44	0.31	0.28–0.39
Destroyed alveoli	0.56	0.52–0.59	0.46	0.39–0.53	0.64	0.61–0.68
Presence of cells	0.99	0.92–1.00	0.87	0.84–0.94	0.99	0.96–1.00
Presence of cell clustering	0.65	0.61–0.69	0.69	0.62–0.74	0.62	0.56–0.68
Presence of blood vessels	0.99	0.95–1.00	0.99	0.96–1.00	0.99	0.97–1.00

Abbreviations: 95%CI: 95% confidence interval

## Discussion

SPNs are estimated to occur at a rate of 200 per 1000 chest CT in high risk patients, with the prevalence of malignancy varying from 5–70% [15, 16]. Lung cancer screening programs have demonstrated that they represent a large majority of lung cancers identified using low dose CT (70.8%) [12]. This emphasizes the need for minimally invasive techniques which could help to diagnose malignancy and create the opportunity for most patients to undergo curative surgery.

This study first indicates that in vivo confocal imaging of distal lung nodules is accessible as long as they are reached with navigational bronchoscopy using an extended working channel. Here, we confirmed that the upper and posterior segments of both lungs appear difficult to reach using the AlveoFlex® miniprobe because of the stiffness of the tip [4], while this difficulty can be overcome using a 0.6 mm miniprobe without compromising the identification of alveolar structures and abnormal patterns.

Due to the current distribution of lung cancers in the lung, the ability to reach SPNs in the upper lobes and peripheral sites is very important. It has been demonstrated that most primary malignant SPNs are located in the upper lobes while two thirds of metastatic SPNs affect the lower lobes [12, 13]. For a single SPN, upper lobe location increases the likelihood of malignancy [17]. While adenocarcinomas are more often detected in the periphery with subpleural predilection, small, irregular benign, subpleural SPNs are extremely common as well. Therefore, the use of the 0.6 mm miniprobe may prove useful in the future for *in-vivo* micro imaging characterization of peripheral lung nodules.

Here, only malignant SPNs were included in the analysis to reduce the possible different variables obtained from image analyses in different lung diseases. This strategy allowed us to demonstrate that confirmation of the guide sheath catheter location into a malignant nodule is possible when an image of solid pattern is obtained, which could potentially optimize a targeted area for biopsy.

We have also shown that in SPNs with identifiable alveolar structures on pCLE imaging, alveolar wall, elastin fibres, cells and blood vessels were recognized easily using both miniprobes. The images from the CholangioFlex® miniprobe demonstrated that these structures can be identified for both analysis and objective measurements. However, the gauge reduction of the CholangioFlex® miniprobe comes at the expense of image quality and spatial resolution. The CholangioFlex® has only 10,000 optical fibers and a field view of 240μm compared to the AlveoFlex® which consists of 30,000 small optical fibers with field of view of 600 μm. As the CholangioFlex® miniprobes used in this study was modified to display the same depth of focus as the AlveoFlex® (0–50μm), the perception of depth was similar in images obtained from both miniprobes.

Despite the differences in image quality, there were no differences in objective measurements such as axial fibre thickness, cell size and frequency for the presence of cells, cell clustering and blood vessels. However, the long and short axis alveolar diameters were significantly larger in images obtained with the AlveoFlex® miniprobe compared to those obtained with the CholangioFlex®. The main reason for this could be due to the field view of the CholangioFlex® miniprobe which is much smaller compared to the AlveoFlex®. This rendered it more difficult to identify an entire normal-sized alveolar mouth for long and short axis diameter measurements using the CholangioFlex® probe. Meanwhile, blood vessel sizes were significantly larger in images obtained with the CholangioFlex® miniprobe. We have previously published that the AlveolFlex® miniprobe could cause compression effects resulting in minimal imaging distortion which may affect the measurement of blood vessel size [4]. We hypothesised that the smaller diameter of the CholangioFlex® miniprobe might have slithered more easily around blood vessels.

However, as the images comparing the two probes originated from different areas of the lungs, we could not exclude that the differences observed might be due to anatomical variance in the lungs.

As there are currently few reported studies examining the appearance of SPN using pCLE, there is a need for a uniform terminology to standardize pCLE image descriptive criteria [11, 18]. A classification of pCLE for SPNs (the Columbus Classification) has been proposed, however this has not been validated to predict cancer prospectively [18]. We have used the term ‘solid pattern’ to describe the dense appearance observed in the majority of the SPNs. However, the study was not designed to apply such descriptive criteria as predictors for malignancy, but to compare the frequency of detection of these criteria to confirm equivalence for images obtained with both miniprobes.

One limitation of the study is that the same lung nodules were not explored using both probes for direct comparison. This is mostly explained because the 1 mm miniprobe failed to progress into the upper lobe location, which led to the choice between the two miniprobes sizes on the basis of the location of the SPN. Therefore, direct comparisons could not be performed as the same SPNs were not analyzed with both probes. Nevertheless, this initial exploratory study showed that the morphometric and non-morphometric information from pCLE images obtained from both miniprobes were comparable.

The interobserver agreement for the pCLE descriptors of SPNs were very good for both the CholangioFlex® and AlveoFlex® groups albeit the item ‘distorted’ alveoli appeared difficult to differentiate from ‘destroyed alveoli’. Interobserver reliability of in-vivo pCLE imaging of the lungs had also been studied previously in non- cancerous lung diseases showing a high degree of image reliability for pCLE especially when diseased were compared to normal alveoli states [19].

pCLE has only recently emerged as a feasible and safe method for in-vivo real time endomicroscopic images of both the central airways and the distal lungs, and to date, the clinical significance of endomicroscopy as a lone or complimentary approach in the diagnosis of lung diseases remains undetermined. Although it is unlikely that pCLE will be able to replace conventional biopsy for lung cancer, pCLE-guided tissue sampling might improve the accuracy and reduce the number of conventional biopsies required. Furthermore, once standardized pCLE images and terminology have been established for lung cancer and solitary pulmonary nodules, pCLE may lead to an optimized rapid in-vivo diagnosis of neoplastic changes in patients with suspected lung cancer. As an example, we have already reported a case in which we made use of pCLE to aid the placement of a fiducial marker for pre-empt stereotactic radiotherapy in a SPN. In this case report, the technique confirmed a solid pattern and destroyed alveolar network before the histological diagnosis of metastatic malignant melanoma was confirmed [20].

In conclusion, this pilot study demonstrated that pCLE imaging is achievable for SPNs in all lobes of the lungs using either the CholangioFlex® or AlveoFlex® miniprobes. As the findings from this study might be too preliminary for a structured strategy on which probe to be used for lung periphery exploration, we suggest that pCLE performers consider the use of the CholangioFlex® for lesions in the upper lobes which may need more manoueuvrability and length to reach the site of interest. We also suggest that the CholangioFlex® could be used in cases where the AlveoFlex® failed to reach the site of interest as depicted in one case involving the right middle lobe. Although there are few differences in the sizes of some alveolar components between the two miniprobes, the frequency of identification of individual alveolar structures were similar. Moreover, the improved manoeuvrability may offer more accessibility to potentially aid the diagnostic work-up of other lung diseases with upper lobe and peripheral predilections. More prospective studies however are necessary to standardize pCLE descriptive criteria and validate its application in SPNs and peripheral lung diseases.

## Supporting information

S1 File Complete protocol of the NODIVEM trial. (PDF)

S2 File TREND statement checklist. (PDF)

## Abbreviations

CT: computer tomography
pCLE: probe-based confocal laser endomicroscopy
r-EBUS: radial probe ultrasound endobronchoscopy
SPN: solitary pulmonary nodule
