# Supplementary material for: Assessing the feasibility of confocal laser endomicroscopy in solitary pulmonary nodules for different part of the lungs, using either 0.6 or 1.4 mm probes
Source: PLoS One. 2017 Dec 21;12(12):e0189846. doi: 10.1371/journal.pone.0189846 (PMC5739462; doi:10.1371/journal.pone.0189846)
Supplement: S1 File — (PDF) [file pone.0189846.s001.pdf]

Title of the study – Assessment of probe based confocal laser endomicroscopy for in-vivo diagnosis of peripheral lung nodules and masses.

"NODIVEM" study. (Ethical Committee approval : CPP NO 2011/030, October 21th 2011)

With the increasing use of chest CT scan in high risk subjects and its ability to detect early stage peripheral lung cancer, solitary pulmonary nodule diagnosis has become a major challenge in respiratory clinical practice. Their management is difficult and, according to published guidelines, often requires close CT follow up, PET CT and invasive procedures to obtain a definite histology.
In this context, innovative endoscopic techniques such as electromagnetic navigation and endoscopic ultrasound using radial miniprobes (EBUS) - also referred as navigational bronchoscopy – have proved to be efficient, in the localization and sampling of peripheral lung nodules that are superior to 20 mm in size. However, none of these techniques is able to differentiate malignant lesions from benign ones, in-vivo, in real time. We have recently developpped the technique of confocal endomicroscopy of the distal lung –also referred as distal lung pCLE or "Alveoscopy" - and published the first images of confocal endoscopy of the normal and tumoral distal lung acquired in vivo.
In this prospective trial we will assess confocal endoscopy as a tool to localize the peripheral lung nodules and to differentiate benign from tumoral lesions.

Objective(s) of the clinical study

1. To demonstrate that confocal endomicroscopy is not inferior to navigational endoscopy for the localisation of peripheral lung nodule
2. To demonstrate that confocal endoscopy can differentiate benign from malignant tumors

Main assessment criteria:

1. number of lesions localized by confocal endoscopy compared to navigational bronchoscopy
2. Positive and negative predictive values of confocal signs before and after IV fluorophore injection for the diagnosis of cancer.

Experimental plan:

Multicentric prospective controlled trial, conducted in three academic centers, specialized in interventional bronchoscopy, equiped with both navigational bronchoscopy and probe based confocal microendoscopy techniques.
Subjects with peripheral lung nodule requiring navigational bronchoscopy will be included in the study and explored using both Confocal endoscopy AND navigational bronchoscopy. Confocal probe will be inserted in the same catheter as used for the navigational bronchoscopy and confocal images will be recorded before and after fluorescein IV injection, and before sampling.

In this this study, positive diagnostic criteria for the localization of peripheral nodule using confocal imaging is a solid pattern as opposed to the usual loose fibered network pattern of the normal alveolar duct. According to this criteria, the confocal imaging procedure will be considered as valid if the number of lesions localized by confocal is not inferior to 90 % of those localized by navigational bronchoscopy. Therefore, pCLE will prove valid if it can recognize at least 70 subjects out of the 78 / 120 subjects for which the navigational bronchoscopy is forecasted to localize the peripheral lesion (Cohen concordance Kappa value of 0,74 between confocal and navigational bronchoscopy).

A secondary objective will be to describe specific signs of cancer using confocal endoscopy . This will be assessed on the first 30 patients using confocal imaging obtained before and after fluorophore IV injection, in comparison to histology, and confirmed on the following 48 patients.

2.6 Clinical research project, English version

This part will include:

- Problem, hypothesis and objective of the investigations ·
- Location of the work in the context of the current knowledge ·
- detailed description of the methodology (number of necessary subjects:)
- detailed description of the implemented techniques ·
- Plan of realization of the describing project:
    - the role of every team
    - a projected calendar adapted to the duration of the project -
    - the identification of the key stages,
    - the modalities of coordination of the project
    - the capacity of patients' inclusion

Synopsis :

The aim of this trial is to assess probe based confocal microendoscopy of the lung (Alveoscopy) for in-vivo diagnosis of peripheral lung nodules and masses, in comparison to modern endoscopic techniques of peripheral lung nodule localisation and sampling (endobronchial ultrasound : EBUS, and electromagnetic navigation : EMN).

Objective(s) of the clinical study

1. To demonstrate that confocal endomicroscopy is not inferior to navigational endoscopy for the localisation of peripheral lung nodule
2. To demonstrate that confocal endoscopy can differentiate benign from malignant tumors

Main assessment criteria:

1. number of lesions localized by confocal endoscopy compared to navigational bronchoscopy
2. Positive and negative predictive values of confocal signs before and after IV fluorophore injection for the diagnosis of cancer.

Experimental plan:

Multicentric prospective controlled trial, conducted in three academic centers, specialized in interventional bronchoscopy, equiped with both navigational bronchoscopy and probe based confocal microendoscopy techniques.

120 subjects with peripheral lung nodule requiring navigational bronchoscopy will be included in the study and explored using both Confocal endoscopy AND navigational bronchoscopy. Confocal probe will be inserted in the same catheter as used for the navigational bronchoscopy and confocal images will be recorded before and after fluorescein IV injection, and before sampling.

Statistical evaluation :

In this this study, positive diagnostic criteria for the localization of peripheral nodule using confocal imaging is a solid pattern as opposed to the usual loose fibered network pattern of the normal alveolar duct. According to this criteria, the confocal imaging procedure will be considered as valid if the number of lesions recognized by confocal is not inferior to 90 % of those localized by navigational bronchoscopy. Therefore, pCLE will prove valid if it can recognize at least 71 of 78 nodules / subjects efficiently sampled using navigational bronchoscopy (Cohen concordance Kappa value of 0,74 between confocal and navigational bronchoscopy).

A secondary objective will be to describe specific signs of cancer using confocal endoscopy . This will be assessed on the first 30 patients (including 20 cancers) using confocal imaging obtained before and after fluorophore IV injection, in comparison to histology, and confirmed on the following 48 patients.

Detailed research project :

I. Problem, hypothesis and objectives of the investigations

I.1 . Peripheral lung nodules

Peripheral lung nodules are an increasing motive of consultation in respiratory medicine (1, 2). This is partly due to the increasing part of peripheral lung adenocarcinoma among primary lung cancers in developped countries (3), and partly to the increasing use of Chest CT scan in asymptomatic high risk subjects.

Chest CT has been used for early lung cancer detection programs in both North America and Japan for more than a decade. (4). These programs have showed a very high frequency of peripheral lung nodules in North America population, since nearly 20 % of the explored subjects have one or several small nodules on baseline screening CT. In USA, 50 % of 50 years old smokers may have an infracentimetric nodule on chest CT (5). From these lesions, identification of malignant nodules is important because they represent a potential curable form of cancer. Whereas the prevalence of malignancy may vary among studies, the vast majority of the lesions smaller than 8 mm are benign, mostly granulomas and harmartoma. In screening studies, the prevalence of malignancy ranges between 2 to 13% of the patients with nodules. In contrast, PET CT studies of lesions larger than 2 cm have shown a a prevalence of 48 to 82% of cancers (1). This underlines the need for a rigourous diagnostic strategy of peripheral lung nodules, according to the size of the lesion and the estimated subject's risk for lung cancer (4).

Until recently there was no scientific validation of CT screening of lung cancer (6, 7). However, the recent release of American NCI sponsored NSLT trial results (8), may impact the clinical practice in a near future. Early results of this randomized trial indicate "twenty percent fewer lung cancer deaths seen among those who were screened with low-dose spiral CT than with chest X-ray". If confirmed, these data will imply that in industrialized countries, the number of asymptomatic subjects undergoing a chest CT scan will increase significantly, so will do the number of patients presenting with a peripheral lung nodule in daily practice

Both Fleichner Society and ACCP have provided guidelines for the management of pulmonary nodules (1, 9), which give a large place for repeated follow-up CT imaging procedures and PET-CT, before taking the decision of histology sampling. In North America, compliance to these guidelines appears acceptable (10). PET-CT false negatives are well known, including resolution limits of the nuclear imaging devices or a low metabolic activity in some cancer lesions such as bronchiolo-alveolar carcinomas and carcinoid tumors. Besides chronic lung bacterial infections (Nocardia), mycobacterial diseases, aspergillosis, sarcoidosis and pulmonary vasculitis are well kown causes of false positive PET-CT results. Because positive and negative predictive value of PET – CT is only 75%, it is generally admitted that a positive PET CT needs histology sampling to be performed, whereas a negative PET –CT requires imaging follow up for two years. (11). However, due to high probability of lung cancer, non calcified nodules larger than two cm are usually biopsied.

I. 2. Endoscopic diagnosis and sampling of peripheral pulmonary nodules.

The histology of a peripheral nodule larger than 1cm can generally be obtained before surgical resection, either by CT guided transthoracic biopsy (TTB) or endoscopically. Recent endoscopic techniques, referred as

navigational bronchoscopy, have the ability to localize and sample peripheral lung nodules larger than 1 cm in size (12). Studies have demonstrated that these techniques have a rentability that is equivalent to transthoracic biopsy sampling but a smaller risk of pneumothorax. (12). The endoscopic procedure for peripheral lung nodule exploration is preferable to TTB in patients suffering from severe respiratory insufficiency, where a iatrogenic pneumothorax may be life threatening, or if the nodule is situated in a central position or at more than 1 cm from the pleura.

Navigational bronchoscopy takes advantage of computering advances in CT image reconstruction, such as virtual bronchoscopy (2). In this case, bronchoscopy is preceeded by reconstruction of segmental and subsegmental inner bronchial surface,computed from the high resolution CT scan. Using this technique, the precise bronchial and segmental pathway (or road map) to the tumor can be forecasted before the endoscopic procedure. Two recent techniques, endobronchial ultrasound using high frequency radial probe (EBUS), and electromagnetic navigation (EMN) have been developped in order to localize peripheral nodules and masses during bronchoscopy. Both devices can be associated with virtual bronchoscopy (12), and make use of the extended working channel (EWC) or guide sheeth technique. EWC is a catheter that surrounds the locatable guide of EMN or EBUS miniprobe and can be extended with it through the bronchoscope working channel down to the lesion to sample. Once the peripheral lesion localized, the probe can be removed, leaving the EWC close to the tumor, through which can be introduced a biopsy forceps or a cytology brush. (13, 14).

Localisation of the nodule is not always easy, depending on the presence of a bronchial sign on CT. Characteristic images can be obtained in real time using EBUS when the nodule is reached (figure 1) .

EMN makes use of a steerable probe containing an electromagnetic sensor. A specific board inserted on the back of the patient’s thorax, produces an electromagnetic field. In this technique, sensor location can be determined in real time within this field , based on Chest CT data and coordonates of known anatomic structures such as main carinas of large bronchi. (figure 2). This technique makes it possible to progress down to the nodule / target with a precision of 2-3 mm. (15, 16).

A randomized trial comparing both EBUS / EMN and their association has shown that each technique has a sensitivity of 65 % for the localisation and diagnosis of peripheral lung nodule, independently from their size (smaller or larger than 2cm) . These techniques do not appear to be strictly equivalent as EBUS provides better results for the nodules located in the lower lobes, that are more mobile, and EMN better results in the upper lobes. In this trial the association of both technique did improve the performance of the navigational endoscopy (80% succes). Owing to the related costs of the technique, an strategy could be to make use of EMN for the upper lobe lesions while using EBUS for the lower lobes.

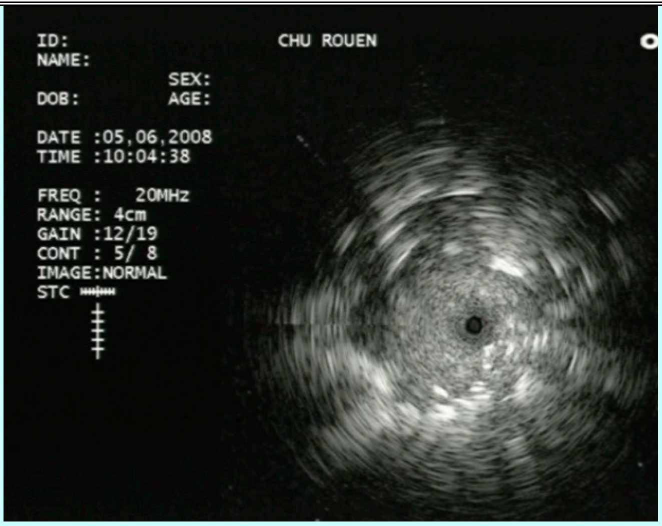

Figure 1 : EBUS imaging of a 1 cm peripheral lung cancer. (Pr L. Thiberville, Rouen University Hospital)

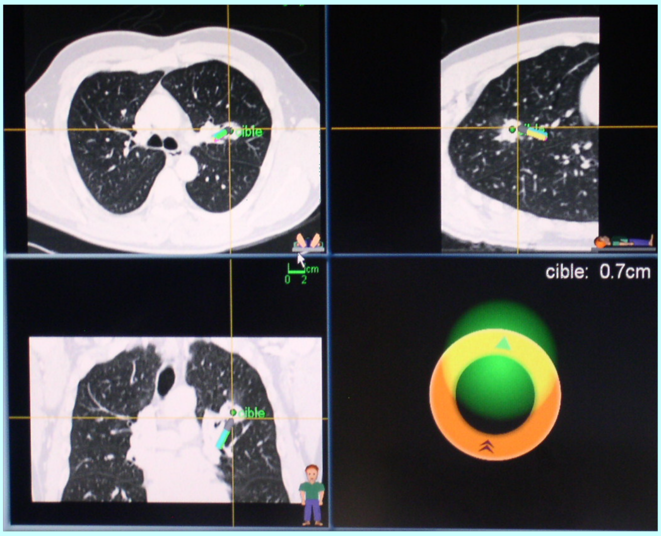

Figure 2: Electromagnetic navigation.

Real time visualisation of the sensor probe progression to the tumor visualized on axial, saggital and coronal CT scan reconstructions. Lower right panel , real time control of the distance from the center of the target. (Pr JM Vergnon, CHU de Saint Etienne)

A noticeable advantage of navigational bronchoscopy is the possibility to place fiducial gold markers within or close to the tumor during bronchoscopy. These radio-opaque markers are highly useful combined with robotized stereotactic radiation therapy techniques such as with Cyberknife, which may be the only available treatment of peripheral lung cancer in patient with advanced respiratory insufficiency.

The main current limitations of navigational bronchoscopy is that the technique cannot differentiate malignant from benign tumors in real time and the low precision for the localisation of the lesion, which is only centimetric.

In this trial we propose to assess a new optical device, called probe based confocal laser endomicroscopy, for the diagnosis of peripheral lung nodule, in comparison with EBUS or EMN.

3. In-vivo imaging of peripheral lung diseases and nodules using probe based confocal laser endomicroscopy.

Probe based confocal laser endomicroscopy (pCLE) of the distal lung is a new optical technology based on the principle of confocal microscopy where the objective has been replaced by a thin (1mm) fiber-bundle or miniprobe.

For in-vivo imaging, the confocal miniprobe is introduced through the working channel of the endoscope and gently advanced down to the tissue to obtain microscopic imaging of the fluorescent structures, in response to 488nm blue light tissue excitation. pCLE makes it possible to visualize microscopic structures 3 µm in size in real time, for a field of view of 600 µm and depth imaging of 50µm. pCLE is currently commercially provided by Mauna Kea Technologies (Paris, France), as a medical device called Cell-Vizio.

Specific optical miniprobes devoted to the exploration of the lung have been first developped at the Rouen University hospital in 2006, where the technique of distal lung imaging has been set up and the first confocal endomicroscopic images of both proximal bronchi and alveoli could be obtained (17, 18). We have shown that Cellvizio makes it possible to prolonge the endoscopic vision to the most distal parts of the lungs, i.e. terminal and respiratory bronchioles as well as alveolar ducts and sacs. (19).

Since 2006, in vivo confocal imaging of the distal lung (a procedure also called Alveoscopy) has been used at the Rouen University – Pulmonology Clinic, in nearly 200 patients as part of a prospective clinical trial (Alveole trial, ClinicalTrial.gov identifier: NCT00213603).

During this trial, we have shown that the technique makes it possible to image both the elastin network of the bronchial wall in vivo (20), and the interstitial elastic fibered network of the distal lung. (19). We have described the optical appearance of normal alveolar ducts and sacs in-vivo in both non smoking subjects and active smokers (figure 3) and showed the perfect tolerance of the technique in awake, spontaneously breathing patients and healthy volunteers. In smokers, Alveoscopy makes it possible to image the cellular alveolar infiltration

from activated fluorescent macrophages. (figure 3) (19). This minimally invasive technique produces very specific images in real time in diffuse interstitial lung diseases such as lung alveolar proteinosis (21).

In a preliminary study (22) we have shown significant differences in the elastic network organisation of the distal lung between healthy controls and patients suffering from lung fibrosis, asbestosis and silicosis, as well as sarcoidosis.

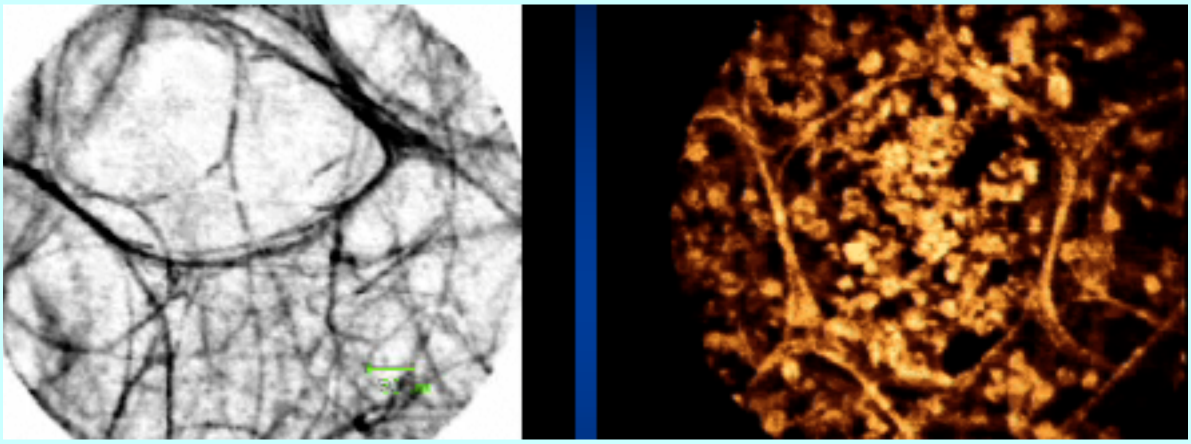

In vivo alveoscopy, non smoker　　　In-vivo alveoscopy, heavy smoker

Figure 3. In vivo alveoscopy of healthy controls *(CHU de Rouen, protocole Alvéole).*

Left panel, normal elastic network imaging in a non smoking subject, right panel : macrophage alveolar infiltration in a smoker. .

Recent data from our group indicates that Alveoscopy *has the potential to provide in-vivo microscopic imaging of peripheral lung nodules and cancers in real time*. (23). This is made possible without exogeneous fluorophore, because of the alterations of the fluorescent elastic network in the tumor tissue. The visualisation of the tumoral cells is also possible after in situ disposition of non-toxic cellular fluorophores such as methylene blue and 660 nm excitation. Figure 4. (23-26).

Before being implemented into clinical practice, our results must be first confirmed from an independant series of patients. In this multicentric trial, we propose to evaluate Alveoscopy, for the diagnosis of peripheral lung nodules, in vivo, in real time. For this purpose, we aim to compare Alveoscopy results with those obtained in the same patients using navigational bronchoscopy techniques.

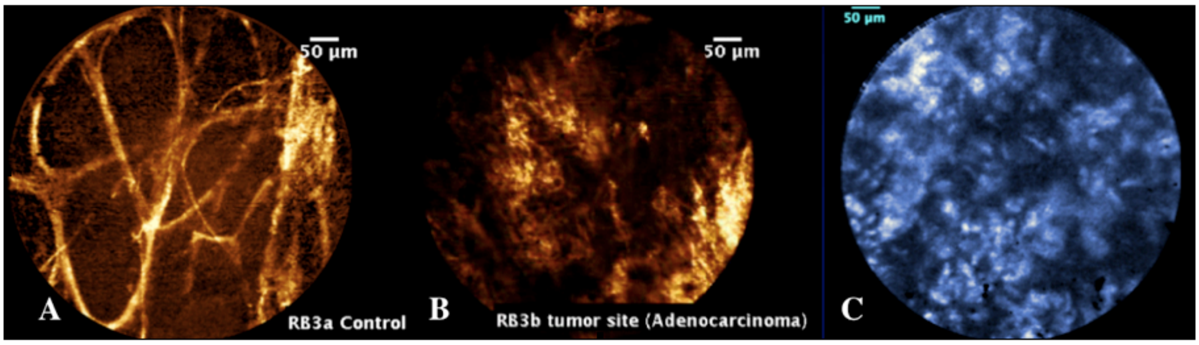

Figure 4 . In vivo confocal imaging of a normal alveoli and a peripheral lung adenocarcinoma (Cellvizio).
A. Normal alveolar duct 488 nm excitation. B. desorganisation of the distal elastic network inside the nodule 488 nm excitation C. Invivo confocal cellular imaging of the nodule after in situ instillation of methylen blue 660 nm excitation. (from Thiberville et al. (23))

Our hypotheses are :

(a), pCLE has the same sentivity than modern navigational endoscopic techniques such as EBUS or EMN for the diagnosis of peripheral lung nodules.

(b) pCLE is able to differentiate in vivo in real time malignant nodules from benign lesions.

The objectives of this study are to assess the ability of pCLE for the precise localization of peripheral lung nodules, and to compare this ability with performances of EBUS or EMN navigation techniques. In this trial, pCLE imaging will considered as successful, if a « solid microscopic patern » can be obtained in vivo, and a diagnosis obtained from the biopsy. These datas will be compared to those of navigation bronchoscopy in the same subject. Positivity criteria for navigational bronchoscopy will be : positive (or contributive) biopsy AND characteristic image using EBUS or progression of the EMN steering probe to less than 5mm of the tumor.

References:

1. Gould MK, Fletcher J, Iannettoni MD, Lynch WR, Midthun DE, Naidich DP, Ost DE. Evaluation of patients with pulmonary nodules: When is it lung cancer?: Accp evidence-based clinical practice guidelines (2nd edition). *Chest* 2007;132:108S-130S.

2. Thiberville L, Salaun M, Lachkar S. [new diagnostic tools in lung cancers]. *Rev Prat* 2009;59:925-931.

3. Pelosi G, Sonzogni A, Veronesi G, De Camilli E, Maisonneuve P, Spaggiari L, Manzotti M, Masullo M, Taliento G, Fumagalli C, et al. Pathologic and molecular features of screening low-dose computed tomography (ldct)-detected lung cancer: A baseline and 2-year repeat study. *Lung Cancer* 2008;62:202-214.

4. Henschke CI, Yankelevitz D, Smith JP, Miettinen OS. Computed tomography screening for lung cancer. *JAMA* 2007;298:514-515; author reply 515-516.

5. Henschke CI, Yankelevitz DF. Ct screening for lung cancer: Update 2007. *Oncologist* 2008;13:65-78.

6. Henschke CI, Shaham D, Yankelevitz DF, Altorki NK. Ct screening for lung cancer: Past and ongoing studies. *Semin Thorac Cardiovasc Surg* 2005;17:99-106.

7. Thiberville L, Corne F. Tumeurs primitives et secondaires du poumon. *Rev Prat* 2004;54:2065-2077; quiz 2077.

8. NCI U. Lung cancer trial results show mortality benefit with low-dose ct. 2010. Available from: http://www.cancer.gov/newscenter/pressreleases/NLSTresultsRelease.

9. MacMahon H, Austin JH, Gamsu G, Herold CJ, Jett JR, Naidich DP, Patz EF, Jr., Swensen SJ. Guidelines for management of small pulmonary nodules detected on ct scans: A statement from the fleischner society. *Radiology* 2005;237:395-400.

10. MacMahon H. Compliance with fleischner society guidelines for management of lung nodules: Lessons and opportunities. *Radiology*;255:14-15.

11. Vaylet F, Bonnichon A, Salles Y, Gontier E, Bonardel G, Lefloch H, Mairovitz A, Mantzarides M, Niang A, Marotel C, et al. La tomographie par emission de positons au 18fluorodesoxyglucose (18fdg-tep) dans la prise en charge du cancer bronchique non a petites cellules en 2006. *Cancer Radiother* 2007;11:16-22.

12. Edell E, Krier-Morrow D. Navigational bronchoscopy: Overview of technology and practical considerations--new current procedural terminology codes effective 2010. *Chest*;137:450-454.

13. Eberhardt R, Anantham D, Ernst A, Feller-Kopman D, Herth F. Multimodality bronchoscopic diagnosis of peripheral lung lesions: A randomized controlled trial. *Am J Respir Crit Care Med* 2007;176:36-41.

14. Kurimoto N, Miyazawa T, Okimasa S, Maeda A, Oiwa H, Miyazu Y, Murayama M. Endobronchial ultrasonography using a guide sheath increases the ability to diagnose peripheral pulmonary lesions endoscopically. *Chest* 2004;126:959-965.

15. Gildea TR, Mazzone PJ, Karnak D, Meziane M, Mehta AC. Electromagnetic navigation diagnostic bronchoscopy: A prospective study. *Am J Respir Crit Care Med* 2006;174:982-989.

16. Bertoletti L, Robert A, Cottier M, Chambonniere ML, Vergnon JM. Accuracy and feasibility of electromagnetic navigated bronchoscopy under nitrous oxide sedation for pulmonary peripheral opacities: An outpatient study. *Respiration* 2009;78:293-300.

17. Salaun M, Bourg-Heckly G, Thiberville L. [confocal endomicroscopy of the lung: From the bronchus to the alveolus]. *Rev Mal Respir*;27:579-588.

18. Thiberville L, Salaun M, Lachkar S, Dominique S, Moreno-Swirc S, Vever-Bizet C, Bourg-Heckly G. Confocal fluorescence endomicroscopy of the human airways. *Proc Am Thorac Soc* 2009;6:444-449.

19. Thiberville L, Salaun M, Lachkar S, Dominique S, Moreno-Swirc S, Vever-Bizet C, Bourg-Heckly G. Human in vivo fluorescence microimaging of the alveolar ducts and sacs during bronchoscopy. *Eur Respir J* 2009;33:974-985.

20. Thiberville L, Moreno-Swirc S, Vercauteren T, Peltier E, Cave C, Bourg Heckly G. In vivo imaging of the bronchial wall microstructure using fibered confocal fluorescence microscopy. *Am J Respir Crit Care Med* 2007;175:22-31.

21. Salaun M, Roussel F, Hauss PA, Lachkar S, Thiberville L. In vivo imaging of pulmonary alveolar proteinosis using confocal endomicroscopy. *Eur Respir J*;36:451-453.

22. Salaun M, Genevois A, Dominique S, Jounieaux V, Zalcman G, Bourg Heckly G, Lachkar S, Hauss PA, Thiberville L. Distal lung elastic network alterations in pulmonary fibrosis, a prospective controlled study using in-vivo confocal endomicroscopy. European Respiratory Society. Barcelona; 2010.

23. Thiberville L, Salaün M, Lachkar S, Dominique S, Moreno-Swirc S, Vever-Bizet C, Bourg Heckly G. In-vivo confocal fluorescence endomicroscopy of lung cancer. *J Thorac Oncol* 2009;4:S49-S51.

24. Thiberville L, Salaun M. Bronchoscopic advances: On the way to the cells. *Respiration*;79:441-449.

25. Thiberville L, Salaün M, Moreno-Swirc S, Bourg Heckly G. In vivo endoscopic microimaging of the bronchial epithelial layer using 660 nm fibered confocal fluorescence microscopy and topical methylene blue. Eur Resp Society Meeting. Stockolm: Eur Resp J; 2007. p. 712S.

26. Thiberville L, Salaün M, Lachkar S, Moreno-Swirc S, Bourg-Heckly G. In-vivo confocal endomicroscopy of peripheral lung nodules using 488nm / 660 nm induced fluorescence and topical methylene blue (abstract). In: J ER, editor. European Respiratory Meeting. Berlin: Eur Resp J; 2008. p. 263s.

II. Méthods

II. 1. General organisation of the research project.

Prospective, multicentric, open-labeled study, assessing a new optical technology for the diagnosis of peripheral lung nodules, in vivo, in real tilme.

II.1.1 Patients and procedures:

Patients with peripheral lung nodule requiring biopsy sampling by navigational bronchoscopy (EBUS or EMN) will be recruited in three French centers : (Pr Thiberville - CHU de Rouen, Pr Vergnon - CHU de Saint Etienne, Dr Hermant - CHU de Toulouse). These three centers are currently equiped with EBUS , Electromagnetic navigation and probe based confocal microendoscopy (Cell Vizio)
During the procedure, at least one normal alveolar area will be imaged using pCLE on the same side than the peripheral lung nodule to serve as normal control. After a successful tentative of nodule localisation using navigational bronchoscopy, the cellvizio miniprobe will be introduced into the extended working channel for confocal in situ imaging. If confocal imaging fails to show a solid network pattern, a biopsy will be performed and the investigator will be autorized to move the navigational device (EBUS or EMN) to another position, followed by another confocal imaging and biospy. A maximum of three attempts to obtain a solid pattern using confocal will be authorized in each patient, after what the procedure will be considered as « failed ». When a solid confocal pattern is obtained, an IV injection of 5 ml fluoresceine sodium 1% will be performed for another imaging before biopsy sampling.

In each case, confocal images will be annoted with the precise identification of the biopsy performed, the images will be stored and a digital copy sent to the Rouen University Hospital for further analysis.

II.1.2. Patient Classifcation :

Before statistical analysis, all the patient charts will be reviewed at 6 months follow up in order to ascertain the definite diagnosis of the nodule. This panel review will rassemble the 3 investigators and a radiologist from one of the center. Panel's analysis will include clinical, histological, radiological as well as any pertinent data on nodule characteristics that may prove to influence the diagnostic yield of the procedures (such as size, localisation, bronchus or vessel convergence sign)

II.1.3. Alveoscopy sequences analysis

Final detailed analysis of alveoscopy sequences will be performed at the Rouen University Hospital, by an independant observer, blindly from the histology results. For this purpose, several items will be analyzed and

coded such as general organisation of the elastin network, solid pattern, vascular network organisation after fluorescein IV injection, or any other items that would appear significant to differentiate benign from malignant lesions.

III. Research trial

III. 1. Hypotheses

(a), pCLE has the same sentivity than modern navigational endoscopic techniques such as EBUS or EMN for the diagnosis of peripheral lung nodules.

(b) pCLE is able to differentiate in vivo in real time malignant nodules from benign lesions.

III.2. Objectives

III.2.1. Primary objective

To determine if Alveoscopy can help to the localization and sampling of a peripheral lung nodule in vivo in showing a solid pattern.

III.2.2. Secondary objectives

a. To determine Sensitivity, specificity, positive and negative predictive values of alveoscopy signs for the differentiation between benign and malignant nodules.
b. To confirm the good tolerance of the technique

III.3. Patient number, statistical analysis

III.3.1. Principal criteria analysis :

In this study, we will consider that, to be valid for the diagnosis od peripheral nodule, alveoscopy should recognize 90% or more nodules that are successfully localized and sampled using navigational bronchoscopy techniques.

Positivity criteria for alveoscopy is defined as in-vivo imaging of a solid pattern and a conclusive biopsy sampling (benign or malignant) of the lesion during one of the navigational attempt to localize the lesion. Alveoscopy will be considered negative if a normal pattern is observed, with a conclusive biopsy of the nodule in the same location.

Navigational bronchoscopy results will be considered positive in case of conclusive biopsy at the first attempt of localisation of the lesion during bronchoscopy.

Therefore, there is a possibility that final results of the study show superiority of confocal imaging over navigational bronchoscopy , if pCLE allows for a better control and correction of the position of the navigational probe during the procedure.

III.3.2. Number of subjects.

Because each patient is evaluated by both navigational bronchoscopy and confocal procedure, the required number of patient can be calculated based on a non-inferiority test for paired data. (Tango T. Equivalence test and confidence interval for the difference in proportions for the paired-sample design, Statistics in medicine 17, 891-908, 1998.) . According to this test, 78 patients positive for navigational bronchoscopy are needed to show a 10 % performance difference between the techniques (alpha risk 5%, Power 80%). This proportion corresponds to a 0,74 concordance using Cohen Kappa test. Forecasted sensitivity of navigational bronchoscopy being 65%, the total number of patients required for the study is 120.

III.3.3. Secondary criteria analysis :

Positive predictive value of confocal imaging for the differenciation of benign versus malignant lesion will be calculated based on histology of the lesion and pertinent in vivo imaging items that will be describe during the study. This semiology will be described from the first 30 patients (including 20 malignant lesions) among the 78 patients for which a definite diagnosis is obtained by the procedure.
The value of this semiology will be confirmed on the 48 further patients.

IV. Realization of the study

IV.1. Location of the research, investigators and authorizations

The endoscopy procedures will be performed by principal investigators in each center (Pr L. Thiberville, Rouen University Hospital) , (Pr Vergnon, St Etienne University Hospital) (Dr Hermant, Toulouse University Hospital). Cell-Vizio Lung device and related « Alveoflex » probe comply to the CE certifications as class Iia medical devices.

Coordination of the study : Pr L.Thiberville, Clinique Pneumologique, CHU de Rouen,
Investigators :

Pr JM Vergnon Service de Pneumologie, CHU de Saint Etienne

Dr C. Hermant, Service de Pneumologie (Pr A. Didier), CHU de Toulouse,

IV.2 Duration of the study, feasibility .

120 patients presenting with a peripheral pulmonary nodule requiring navigational bronchoscopy will be included in the three centers. Patient recruitment will be competitive, during a 30 months period, with a forecasted inclusion rate of one to two patients per center each month. The total duration of the study will be 3 years including 6 month follow up. The choice of the reference navigational technique will be determined by the investigator for each patient before the procedure.

Feasibility of the project relies on :

- The large experience of Pr Thiberville in the confocal technique
- The recognized experience of each investigator in interventional bronchoscopy and navigational bronchoscopy (both EBUS and EMN) with at least 50 procedures already performed in each center, and the fact that the centers are currently equipped with Cell vizio.
- Strong professional links between investigators already exist, as the three investigators have participated to multicentric trials coordinated by Pr Thiberville, such as Seleprebb study (Clinical trial identifier NCT00213603, supported by PHRC 2002) and EVIEPEB study (Clinical trial identifier NCT00960271, supported by INCa 2007). The three investigators are active members of the GELF group (groupe d'endoscopy de langue Française), coordinated by Pr Vergnon.

IV.3. Inclusions

Patients will be prospectively included in each center, according to the French regulations for clinical trials, and after AFSSAPPS authorization and Ethical comity approval of the study. Quality assurance will be provided by the Rouen University Hospital, unique sponsor of the study. Informed consent will be acquired from the patient before the procedure according to Helsinki rules.

Each patient inclusion will be declared in real time to the Rouen University direction of research and innovation by Fax

IV.3.1. Inclusion criteria

- Patients with a peripheral lung nodule accessible by navigational bronchoscopy and cell vizio according to Investigator evaluation of the CT scan.
- 18 years old or more.
- Affiliation to french social security insurance.
- Signed informed consent for the procedure.

IV.3.2. Non-Inclusion criteria

- Apical and dorsal segment lesions of both upper lobes, which are currently not accessible using cell vizio Alveoflex miniprobes.
- Severe respiratory insufficiency that will not allow the bronchoscopy procedure
- Uncorrected bleeding disorders
- History of pneumonectomy or exploration controlateral to a non functional lung,
- Pregnant or breast feeding woman, or person not authorized to participate to a clinical trial according to L1121-6 et L1121-8 of French Code de la santé Publique, concurrent participation to another clinical trial

IV. 4. Endoscopy procedure

- Alveoscopy procedure will be performed according to the procedure validated at the Rouen University Hospital, in genlty advancing the confocal miniprobe through the bronchoscope working channel and distal bronchus until the alveolar fluorescent signal could be obtained. The probe will then stay in place

5 to 30 seconds for alveolar sequence recording

- Navigational bronchoscopy (EBUS / EMN) will be perfored according to the usual validated procedure.
- Confocal imaging of the peripheral nodule will be performed by entering the confocal miniprobe into the extended working channel during the navigational bronchoscopy procedure as described in chapter II.1.1..
- Confocal exploration duration will not exceed 10 minutes for the totality of the endoscopy procedure.
- Usual monitoring (EKG, transcutaneous arterial saturation) will be recorded continuously during the procedure, and chest radiograph performed after the bronchoscopy to control the absence of pneumothorax.
- Patient could be discharged after the procedure after one hour, and physical examination.

IV. 5. Tolerance. Interruption of the study.

Tolerance data will be collected and analyzed prospectivelly. Interruption of the study could be decided by the spon of the study if justified based on this safety data surveillance.

IV.6. Role of each team

The study will be conducted in three academic centers.

1. CHU de Rouen
2. CHU de Toulouse
3. CHU de Saint-Etienne

.

Each Investigator will be responsible for the local recruitement into the study and will conduct the study in his center according to good practice guidelines. All investigators will participate to the central review of the patients' charts

Confocal image analysis for initial description of malignant lesions semiology (secondary objective) will be performed prospectivelly for the 38 first informative cases. These data will analyzed jointly by the three principal investigators in order to describe confocal semiology that could differentiate malignant from benign lesions.

After this step, this confocal semiology will be recorded on the following patients, and data analyzed by an independant observer, blindly from the results of the histology.

IV.7. Duration of the study

Start of the study : september 2011

Inclusion : 30 months

Chart's review and validation : 6 months after the endoscopic procedure

Total duration of the study : 3 years.

V-Ancillary study (submitted to the ethical committee and approved July 20th, 2012 N° approval CPP 2011/30 Amdt 1) .

At the Rouen University Hospital, and Toulouse University Hospital, an ancillary study will be performed in a subset of the study population, using a 0.6 mm modified Cholangioflex® miniprobe in order to explore the nodules in the upper lobe segments. The objectives of the ancillary study are to confirm the possibility to reach the upper lobe, and to compare the imaging performances of this probe to the standard 1 mm Alveoflex® probe.
